# Tailoring the Release of Paclitaxel from Electrospun Nonwovens

**DOI:** 10.3390/ijms262311540

**Published:** 2025-11-28

**Authors:** Bartosz Jaroszewski, Monika Musiał-Kulik, Ryszard Smolarczyk, Tomasz Cichoń, Alina Drzyzga, Ewelina Pilny, Mateusz Stojko, Jakub Włodarczyk, Joanna Jaworska, Anna Kaps, Piotr Paduszyński, Marzena Jaworska-Kik, Małgorzata Pastusiak, Paweł Chaber, Arkadiusz Orchel, Katarzyna Jelonek, Janusz Kasperczyk

**Affiliations:** 1 Department of Biopharmacy, Faculty of Pharmaceutical Sciences in Sosnowiec, Medical University of Silesia, Jedności 8, 41-200 Sosnowiec, Poland; 2 Centre of Polymer and Carbon Materials, Polish Academy of Sciences, Curie-Skłodowska 34 St., 41-819 Zabrze, Poland; 3 Center for Translational Research and Molecular Biology of Cancer, Maria Skłodowska-Curie National Research Institute of Oncology, Gliwice Branch, Wybrzeże Armii Krajowej Street 15, 44-102 Gliwice, Poland

**Keywords:** paclitaxel, implantable drug delivery, local treatment, PLGA, bioresorbable polymer, cancer

## Abstract

Implantable drug delivery devices may enhance therapeutic efficacy by allowing localized drug release, and they may overcome the drawbacks of conventional systemic treatment. Electrospun nanofibers are promising drug delivery systems due to their high surface-to-volume ratio, porosity, and easy drug encapsulation. However, controlled and sustained drug release is required to improve therapeutic efficacy and reduce toxicity. Also, the ability to tailor the release drug dose would be a useful tool for providing an optimal and individualized approach for the treatment. Therefore, the aim of the study was to analyze the possibility to tailor the release of paclitaxel (PTX) from poly(D,L-lactide-co-glycolide) (PDLGA) electrospun nonwovens by modifying the comonomer molar ratio. For this purpose, three kinds of polymers were compared with lactidyl-to-glycolidyl comonomer ratios of 86:14, 70:30, and 48:52. Also, nonwovens obtained from a blend of PDLGA and PVA were used to analyze the effect of the addition of the hydrophilic polymer on degradation and, thus, the release rate. The comprehensive analysis of the developed nonwovens was conducted through an evaluation of the morphology, in vitro degradation, and drug release process, as well as cytotoxicity. It has been observed that all kinds of the developed PDLGA nonwovens provide an extended-release profile but with different release rates, which depend on the comonomer unit ratio and molar mass of the copolymer. Moreover, the increase in hydrophilicity caused by PVA sufficiently accelerates PTX release. The biological activity of released PTX was confirmed under in vitro and in vivo conditions against 4T1 mouse mammary carcinoma. The results of the study enabled us to gain insight into the influence of polymer choice on PTX release from PDLGA ES implants, which may be helpful in their easier translation into the clinic and for better adjustment of the PTX dose for individual treatment.

## 1. Introduction

The World Cancer Report indicates that cancer represents one of the main causes of premature mortality worldwide [1]. Despite the advances in oncology, drug distribution to solid tumors remains a prevailing challenge for effective treatment. Currently, most clinically available oncology drugs with sustained release are administered systemically, which is associated with many adverse side effects [2,3]. Systemic therapies often fail due to difficulties in achieving therapeutic drug levels within the tumor. For example, in the case of intravenously infused paclitaxel (PTX), less than 0.5% of the total dose is locally available within the tumor [4]. In order to limit the common systemic side effects and toxicity, research is currently focused on the development of innovative treatments to specifically target cancer cells [5]. An example of this strategy may be a loco-regional drug delivery system that may overcome transport barriers and improve the therapeutic index and efficacy [4,6]. Formulations and devices, such as wafers, scaffolds, foams, and fibers, have been developed with aim of minimizing local tumor recurrence and reducing detrimental side effects to healthy organs and tissues through loco-regional administration [3,5,7]. Gliadel^®^ wafer was the first delivery system designed for the local treatment of recurrent malignant brain tumors, which was approved by the Food and Drug Administration (FDA) in 1995 [3]. A high therapeutic potential has also been reported for other polymeric patches developed for local cancer treatment. The patches obtained from a mixture of poly(lactide-co-glycolide) (PLGA), polycaprolactone (PCL), and 5-fluorouracil by 3D printing demonstrated in a subcutaneous mouse model of pancreatic cancer that the local drug administration was three times higher compared to the systemic route and did not induce systemic toxicity [8]. Electrospun (ES) nanofibers present an even more promising platform for drug delivery, since they provide a high surface area-to-volume ratio, controllable pore sizes, and a tunable drug release profile [5,9]. Electrospinning constitutes a fiber production method that employs electrical forces to create charged polymer jets, which experience electrically driven bending instabilities and solidify to produce long, continuous nanofibers with diameters typically between tens of nanometers and a few micrometers. The advantage of this technique is the possibility of loading a wide variety of poorly soluble drugs into the fibers to improve their bioavailability or to achieve controlled release [10]. Although none of the electrospun products have been approved by the FDA yet, Nicast obtained a Conformitè Europèenne (CE) certification in 2008 for its AVflo™ product and Biotronik received a CE mark in 2013 for PK Papyrus. There are also several emerging ES products for wound dressing applications that are not commercially available yet but are currently undergoing clinical studies [11].

Anticancer drug-loaded ES nanofibers may enable controlled and sustained drug release at the desired site of action with improved efficacy. They can be used as an implant at the tumor site to prolong the drug release or into a post-operative tumor cavity to inhibit tumor recurrence. The electrospinning method may be applied for the incorporation of commonly used anticancer drugs, such as PTX. PTX, also known as Taxol^®^, derived from the *Taxaceae* family is used to treat breast, ovarian, and lung cancers. It is a microtubule-targeting agent that stabilizes these structures and arrests cells in the G2/M cycle phase, which leads to cell death [12]. Unfortunately, the Cremophor used as a PTX formulation vehicle is not inert and is commonly associated with severe anaphylactoid hypersensitivity reactions, abnormal lipoprotein patterns, hyperlipidemia, erythrocyte aggregation, and peripheral neuropathy [13,14]. Therefore, there have been some attempts to incorporate PTX into polymeric delivery systems, including ES nonwovens [15]. Despite the apparent simplicity of electrospinning, it also holds significant potential for modification and application in combined therapies, e.g., PTX with gold nanorods, PTX and curcumin [16], or nonwovens for the oral delivery of PTX [17]. It is essential to establish a system that provides a controlled and sustained drug release profile. It has been observed that PTX incorporated into a nonwoven composed of chitosan and polyethylene oxide exhibited a significant burst release effect and 80% of the drug was released within the initial 50 h [17]. A prolonged release profile was demonstrated for PTX incorporated into the nonwoven composed of a blend of poly(glycolide-co-ε-caprolactone) and poly(D,L-lactide-co-glycolide). Nonwoven containing 4% of PTX released ≈ 20% of the drug in the first 10 days, but the release process was extended to 84 days [18].

Poly(lactide-co-glycolide) (PLGA), a biodegradable and biocompatible copolymer, is extensively utilized in medical and pharmaceutical applications because it may provide sustained drug delivery, protect pharmaceutical cargo, and be tailored to achieve specific release kinetics [19,20]. PLGA has regulatory approval from the Food and Drug Administration (FDA) and European Medicines Agency (MDA) for drug delivery applications [20]. It can be synthesized from cyclic monomers (lactide (LA) and glycolide (GA)) at various ratios. In general, the polymers with a higher content of lactidyl units provide longer degradation. A higher content of glycolidyl units accelerates degradation due to increase in polymer hydrophilicity [20,21,22]. Since the characteristics of the polymer affect the degradation process, which subsequently has an impact on the kinetics of the drug release, it allows us to tailor the properties of drug delivery systems obtained from PLGA.

These examples demonstrate the great potential and efficacy of ES nonwovens loaded with anticancer drugs and PLGA for the formation of various drug delivery systems. However, there is still a need to develop a method for controlling the drug release rate, which would be helpful in adjusting the drug dose for particular therapeutic requirements. Therefore, the aim of our study was to analyze the effect of selecting poly(D,L-lactide-co-glycolide) (PDLGA) ES nonwovens with different comonomer molar ratios on the release process of PTX. For this purpose, three kinds of polymers with a lactidyl-to-glycolidyl comonomer ratio of 86:14, 70:30, and 48:52 were compared. The composition of PDLGA affects the rate of degradation and, therefore, the kinetics of drug release. Therefore, the modification of the PLDGA composition facilitates the creation of a delivery system that exhibits the desired drug release profile [21]. Additionally, nonwoven obtained from a blend of PDLGA and poly(vinyl alcohol) (PVA) was used to analyze the effect of the introduction of the hydrophilic polymer on degradation and, thus, the release rate, which has not been analyzed so far. It has been already reported that the incorporation of PEG may enhance the release kinetics of PTX from PLA fibers [23]. The comparison of four different ES nonwovens differing in comonomer composition and hydrophilicity may allow us to gain new insight into the influence of polymer choice on tailoring PTX release from PDLGA ES implants.

## 2. Results

### 2.1. Characterization of Electrospun Nonwovens

**Figure 1:**
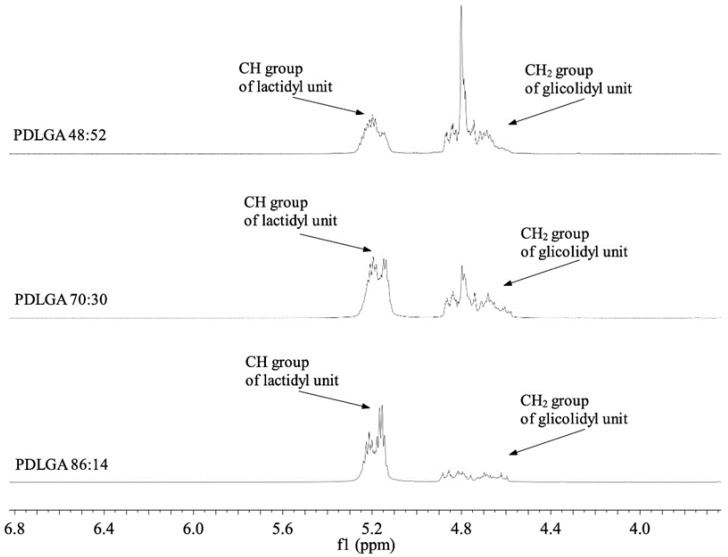
^1^H NMR spectra (600 MHz, DMSO-d_6_) of poly(D,L-lactide–co-glycolide) with various comonomer molar ratios (ethylene and methane region).

Three PDLGA copolymer compositions were synthesized through ring-opening polymerization (ROP). The final compositions of the obtained copolymers were 86:14, 70:30, and 48:52 (lactidyl/glycolidyl comonomer units) (Figure 1).

Three kinds of copolymers with various lactidyl (D,L-LA)-to-glycolidyl (GA) ratios (Figure 1) and PDLGA 48:52/PVA blends were used for the preparation of nonwovens with PTX. The lactidyl-to-glycolidyl unit ratio of the copolymers was 86:14, 70:30, and 48:52, which is close to the feed ratio. The characteristics of the physicochemical properties of the nonwovens is presented in Table 1. The highest molar masses were observed for PDLGA 86:14 + PTX (66.1 kDa) and PDLGA 70:30 + PTX (61.0 kDa). Significantly lower molar mass were observed for PDLGA 48:52 + PTX and PDLGA 48:52/PVA + PTX (29.1 kDa and 25.0 kDa, respectively). The T_g_ of all kinds of nonwovens was above human body temperature (around 50 °C). The T_m_ was between 80–83 °C for the PDLGA 86:14 + PTX, PDLGA 70:30 + PTX and PDLGA 48:52 + PTX, and it was 96 °C in the case of PDLGA 48:52/PVA + PTX.

To evaluate the difference in surface hydrophilicity of PDLGA 48:52 + PTX and PDLGA 48:52/PVA + PTX, the contact angle was measured. As presented in Table 1, the contact angle of the PDLGA 48:52 + PTX nonwoven was higher compared to the contact angle of PDLGA 48:52/PVA + PTX. The contact angle of nonwovens also differed depending on the side, and it was lower on the smoother side that was attached to the collector during the production process.

**Figure 2:**
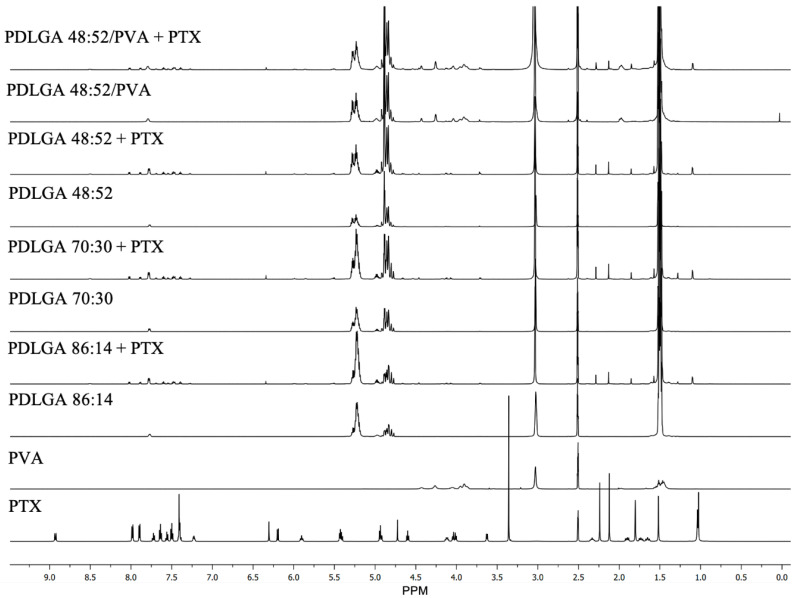
Comparison of ^1^H NMR spectra (600 MHz, DMSO-d_6_) of PTX, drug-free nonwovens, and nonwovens containing PTX.

The successful incorporation of PTX in the nonwovens was visualized in the NMR spectra (Figure 2). In the spectra obtained for all kinds of the drug-loaded ES nonwovens, the signals typical for PTX have been identified, e.g., phenyl groups at 7.35–8.13 ppm. Those peaks were not visible in the spectra obtained for drug-free nonwovens. The dose of PTX per mg of nonwoven was very similar for each kind of material (Table 1).

**Figure 3:**
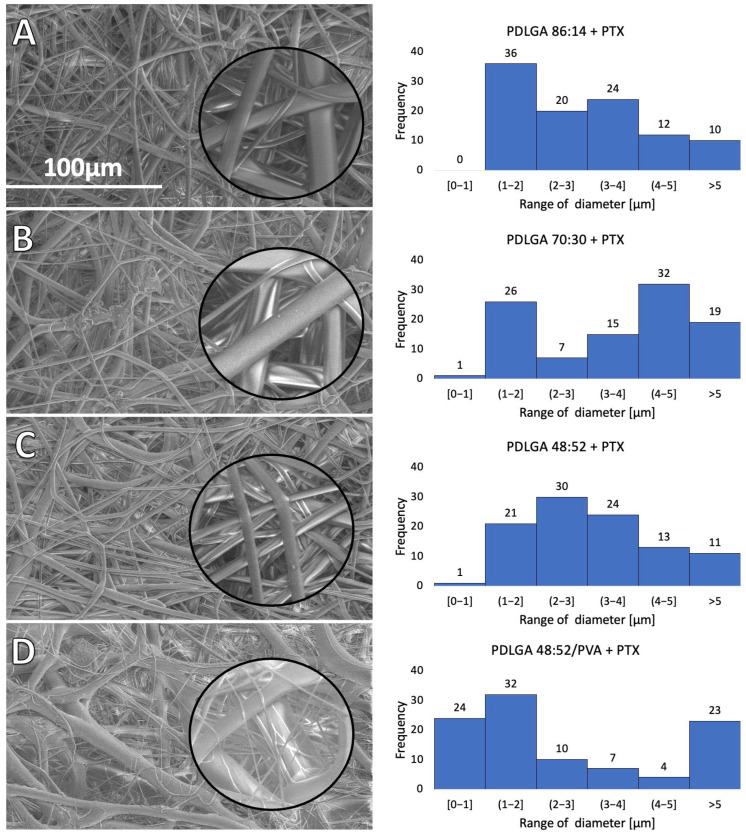
SEM images and histograms presenting the fiber size distribution of nonwovens containing PTX: PDLGA 86:14 + PTX (**A**); PDLGA 70:30 + PTX (**B**); PDLGA 48:52 + PTX (**C**); and PDLGA 48:52/PVA + PTX (**D**). The main images are displayed at a magnification of ×1000, while the close-up regions within the circles are displayed at a magnification of ×5000.

The morphology of the nonwovens was observed by means of SEM (Figure 3), and the ImageJ graphics program was used for measuring the diameter of the fibers (Table 2). All kinds of polymers were suitable to form nonwovens with a regular, smooth fiber surface, without pores, the presence of beads, a ribbon-like appearance, or any other kind of surface irregularities. The average diameter of the fibers was similar for all kinds of polymers–from 2.74 µm for PDLGA 48:52/PVA + PTX to 3.96 µm for PDLGA 70:30 + PTX. Despite the similar diameter of the fibers, the nonwoven obtained from the PDLGA 48:52/PVA + PTX formed a wide range of fiber sizes, from very thin to very thick ones (Figure 3D).

### 2.2. In Vitro Degradation of PDLGA Nonwovens

The degradation of PDLGA nonwovens was studied under in vitro conditions for 168 days. Changes in copolymers were evaluated by analyzing the molar mass, thermal properties, comonomer molar ratio, and weight loss.

**Figure 4:**
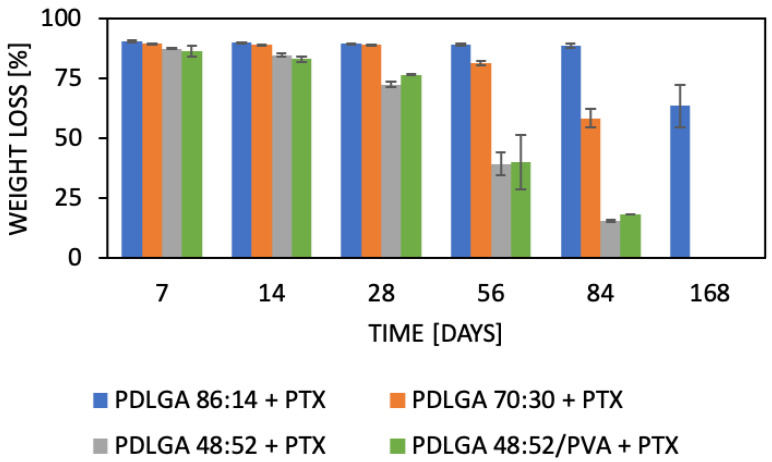
Comparison of weight losses of nonwovens (PDLGA 86:14 + PTX; PDLGA 70:30 + PTX; PDLGA 48:52 + PTX and PDLGA 48:52/PVA + PTX nonwoven) during in vitro degradation (*n* = 3; ±SD).

The changes in weight of the material upon degradation are presented in Figure 4. An insignificant decrease in weight was observed for all kinds of polymers for first 14 days. Beyond that, a constant decrease in the weight of PDLGA 48:52 + PTX was observed up to 72%, 40%, and 15% after 28, 58, and 84 days, respectively. A similar weight loss was observed for PDLGA 48:52/PVA + PTX. After 168 days, both kinds of nonwovens were significantly degraded and disintegrated, so the remaining material was too small for the precise weighting of the sample. A significantly slower weight loss was observed for PDLGA 86:14 + PTX (63% after 168 days) and PDLGA 70:30 + PTX (58% after 84 days). PDLGA 70:30 + PTX disintegrated within 168 days, which made the evaluation of its weight impossible.

**Figure 5:**
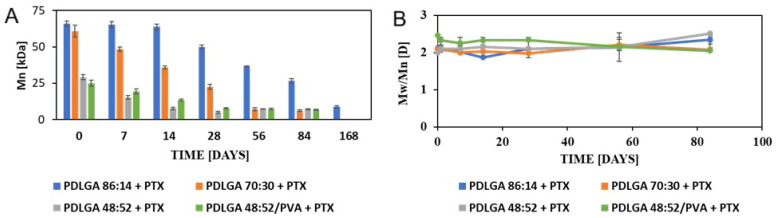
Changes in the number-average molar mass (M_n_) (**A**) and dispersity of molar mass (D) (**B**) of nonwovens containing PTX (PDLGA 86:14 + PTX; PDLGA 70:30 + PTX; PDLGA 48:52 + PTX and PDLGA 48:52/PVA + PTX) during in vitro degradation (*n* = 3; ±SD).

Changes in the M_n_ and molar mass dispersity (D) during degradation were evaluated by means of GPC and are presented in Figure 5A,B, respectively. The slowest change in the M_n_ was observed for PDLGA 86:14 + PTX, which started after 14 days and decreased from 66.1 to 50.1 kDa, 36.6 kDa, 26.6 kDa, and 8.9 kDa after 28, 56, 84, and 168 days, respectively. The decrease in molar mass of the PDLGA 70:30 + PTX (61.0 kDa) proceeded very steadily and after 84 days it was only 6.1 kDa, so beyond this time it mostly degraded, which made it impossible to analyze the sample after 168 days. The M_n_ of PDLGA 48:52 + PTX (29.1 kDa) decreased to 15.3 kDa already after 7 days and to 5.1 kDa after 28 days. The changes in PDLGA 48:52/PVA + PTX proceeded very similarly to the PDLGA 48:52 + PTX. The changes in molar mass dispersity were very insignificant for all kinds of copolymers, as presented in Figure 5B, which is very advantageous for a regular drug release process.

**Figure 6:**
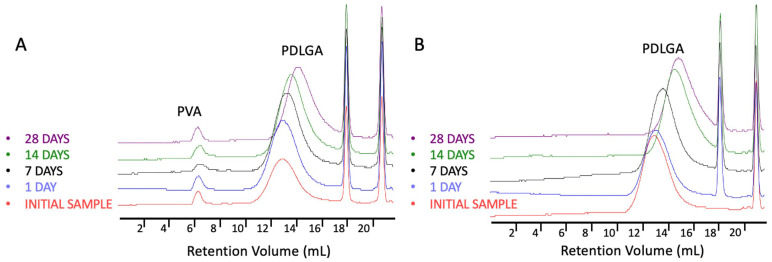
Comparison of GPC chromatograms of PDLGA 48:52/PVA + PTX (**A**) and PDLGA 48:52 + PTX (**B**) after various degradation times.

Additionally, the presence of PVA in PDLGA 48:52/PVA + PTX nonwovens was monitored using GPC chromatograms, as shown in Figure 6. The peaks assigned to the PVA were detected for at least 28 days of degradation, which proves the PVA remains in the nonwovens.

The changes in the ratio of lactidyl to glycolidyl units during degradation were analyzed by ^1^H NMR. An analysis of the changes in comonomer composition is performed to obtain more details about the copolymer during the degradation process [24,25,26]. As presented in Table 3, very insignificant changes were observed for PDLGA 84:16 + PTX and PDLGA 70:30 + PTX, which showed a slight increase in lactidyl and a decrease in glycolidyl units after 84 and 56 days, respectively. The increases in lactidyl unit content in the other two kinds of nonwovens were more significant and began much faster—already after 7 days.

Also, changes in the T_g_ were observed (Table 4); however, it remained above human body temperature for the entire duration of the degradation. The T_g_ is an important physical characteristic of materials [27,28,29]. Amorphous polymers are hard and brittle at temperatures below T_g_. Above the T_g_ they become elastic and, thus, more susceptible to degradation.

**Figure 7:**
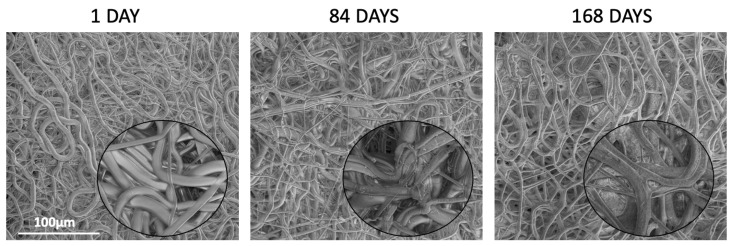
SEM images of the PDLGA 84:16 + PTX nonwoven after 1, 84, and 168 days of degradation. The main images are displayed at a magnification of ×1000, while the close-up regions within the circles are displayed at a magnification of ×5000.

**Figure 8:**
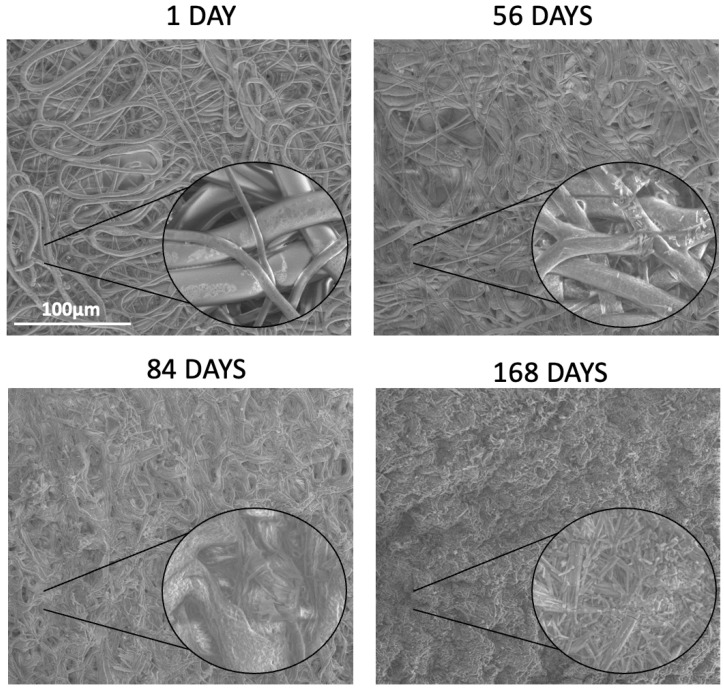
SEM images of the PDLGA 70:30 + PTX nonwoven after 1, 56, 84, and 168 days of degradation. The main images are displayed at a magnification of ×1000, while the close-up regions within the circles are displayed at a magnification of ×5000.

**Figure 9:**
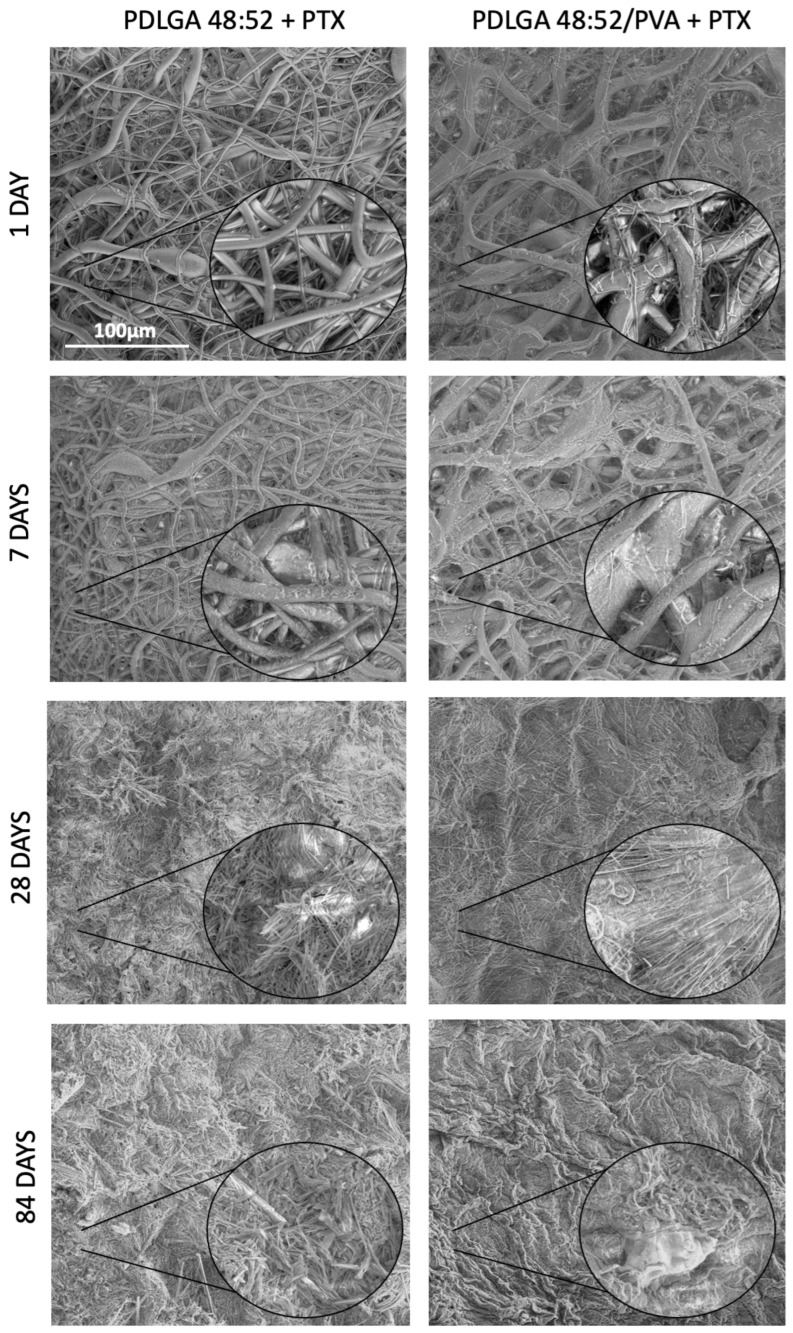
SEM images of the PDLGA 48:52 + PDLGA 48:52/PVA + PTX nonwoven after 1, 7, 28, and 84 days of degradation. The main images are displayed at a magnification of ×1000, while the close-up regions within the circles are displayed at a magnification of ×5000.

During the entire degradation process, the morphology of the nonwovens was observed by means of SEM to determine changes in the fibrous structure, including fiber cracking, deformation, porosity formation, and the disappearance of spaces between the fibers. Additionally, the diameter of the fibers was monitored during the degradation process, and the results are summarized in Table S1. It can be observed that all kinds of nonwovens show a tendency toward a slight increase in the fibers’ diameter, which is caused by water diffusion and swelling. The degradation of the fibers was accompanied by the appearance of PTX crystals on the surface of the fibers. The most stable morphology upon degradation was demonstrated by PDLGA 84:16 + PTX (Figure 7), because only small cracks were observed on the fiber surface after 84 days of degradation. Between 84 and 168 days, degradation slightly accelerated, which caused the formation of pores on the fibers and the presence of PTX crystals on the surface. Similarly, the degradation of PDLGA 70:30 + PTX proceeded regularly, and after 56 days, the fibrous structure was still visible, although the fibers were slightly deformed and covered with PTX crystals (Figure 8). More defined changes were observed after 84 days due to the deformation of the fibers and the significant decrease in spaces between them, leading to a more solid structure after 168 days. The changes in the morphology of the PDLGA 48:52 + PTX and PDLGA 48:52/PVA + PTX nonwovens during degradation proceeded significantly more dynamically (Figure 9). In both kinds of nonwovens, deformed and porous fibers with PTX crystals on their surface already appeared after 7 days of degradation. After 28 days, the fibrous structure disappeared, and the samples became more solid, with visible PTX crystals. The PDLGA 48:52 + PTX and PDLGA 48:52/PVA + PTX nonwovens demonstrated similar morphology also after 84 days.

### 2.3. In Vitro Drug Release 

**Figure 10:**
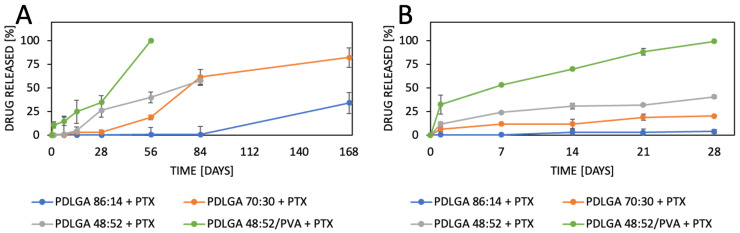
Cumulative release of PTX from various kinds of nonwovens over 168 days of incubation in PBS (**A**) or during 28 days in PBS with Tween 80 (0.5%) (**B**) (*n* = 3).

The release of PTX from the developed nonwovens was analyzed over 168 days in two different media—PBS (Figure 10A) and PBS with Tween 80 (0.5%) (Figure 10B). Significant differences in PTX release from different kinds of nonwovens were observed. The drug release was very slow in PBS. The fastest release was observed from PDLGA 48:52/PVA + PTX, which was the only nonwoven that achieved drug release from the first day (10%), and the process was completed after 56 days. Significantly less drug was released from PDLGA 48:52 + PTX as only 38% was released after 56 days and 55% after 84 days. PTX release from PDLGA 70:30 + PTX nonwovens was initiated after 7 days and reached 75% after 168 days. PDLGA 85:15 + PTX presented the slowest PTX release, with a lag time of 28 days and accelerated release between 84 and 168 days.

The release process conducted in PBS with Tween 80 (0.5%) for 28 days (Figure 10B) presents a much more regular profile. However, the differences between nonwovens are similar to the process observed in PBS—the slowest PTX release rate was observed for PDLGA 85:15 + PTX and the fastest for PDLGA 48:52/PVA + PTX.

The drug release data (Figure 10B) were fitted to kinetic mathematical models (Higuchi, Korsmeyer–Peppas, and Peppas–Sahlin) to determine the mechanism of PTX release (Table 5). The release data present good correlation with the Korsmeyer–Peppas and Peppas–Sahlin model. In the Korsmeyer–Peppas model, the decrease in the value of the exponent (n) is observed along with the decrease in lactidyl unit content in the copolymer, so the n is the highest in PDLGA 86:14 + PTX and the lowest in PDLGA 48:52 + PTX. The n exponent serves as an indicator of the release mechanism, encompassing Fickian diffusion (*n* = 0.5), anomalous transport (0.5 < *n* < 1.0), case II transport (*n* = 1.0), and super case II transport (*n* > 1.0) [30]. The results presented in Table 5 indicate that mainly polymeric behavior controls PTX release from the PDLGA 86:14 + PTX nonwoven. A decrease in lactidyl unit content causes a shift toward diffusion-controlled release. These observations are confirmed by the parameters of the Peppas–Sahlin model. The Pappas-Sahlin model enables us to compare values of the k1 (Fickian diffusion contribution) and k2 (case II relaxational contribution) to describe the dominant mechanism of drug release [31]. Only in the case of PDLGA 86:14 + PTX is k2 > k1, which confirms that the drug release is controlled mainly by the relaxation and swelling of the polymer. Contrarily, in the case of all other three types on nonwovens, the k1 > k2, which proves that Fickian diffusion is the dominant mechanism of drug release. The highest influence of Fickian diffusion was observed for PDLGA 86:14/PVA + PTX.

### 2.4. In Vitro Antiproliferative Activity

**Figure 11:**
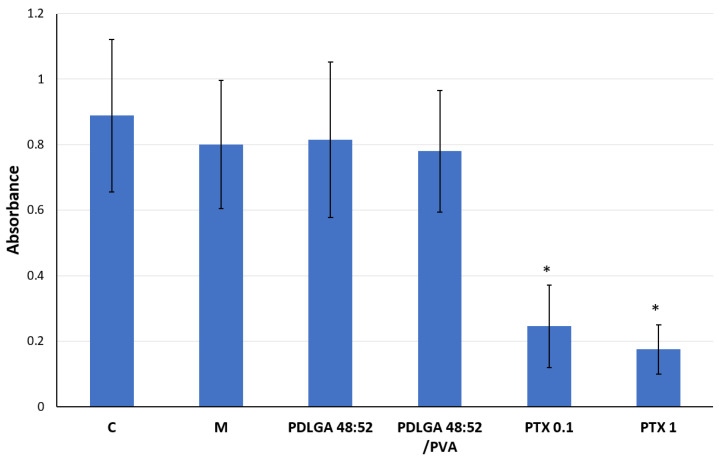
Growth of 4T1 cell line determined using the SRB assay. C—control (fresh medium); M—medium pre-incubated at 37 °C for 24 h (extraction condition); PDLGA 48:52—undiluted extracts from drug-free PDLGA 48:52 nonwoven; PDLGA 48:52/PVA—extracts from drug-free PDLGA 48:52/PVA nonwoven; PTX 0.1—paclitaxel solution at a concentration of 0.1 µg/mL; and PTX 1—paclitaxel solution at a concentration of 1 µg/mL. Each bar represents the mean ± SD; * *p* < 0.05 compared with control (RB-ANOVA); *n* = 4.

To prove the pharmacological activity of PTX released from nonwovens, the antiproliferative effect of extracts obtained from drug-loaded fibers was examined. Additionally, the cytotoxicity of drug-free nonwovens as well as pristine PTX was evaluated for comparison. Two types of ES nonwovens were selected for this biological evaluation as follows: one made from the PDLGA 48:52 copolymer and the other made from a blend of that aliphatic polyester and PVA (PDLGA 48:52/PVA). As shown in Figure 11, under the conditions of the extraction process, the cell culture medium retained its ability to support 4T1 cell viability. It was evidenced by efficient cell proliferation in the medium pre-incubated for 24 h at 37 °C, similarly to the media containing polymeric samples. Both types of the drug-free nonwovens turned out to be completely cytocompatible as they did not cause any substantial inhibition of cell growth. The exposure of the cells to PTX solutions for 72 h resulted in a considerable reduction in their growth.

**Figure 12:**
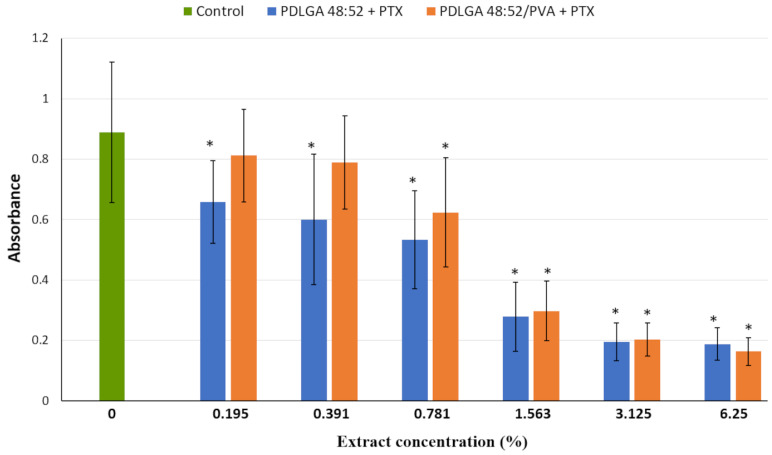
Growth of 4T1 cells in the presence of extracts from PDLGA 48:52 + PTX and PDLGA 48:52/PVA + PTX. Each bar represents the mean ± SD; * *p* < 0.05 compared with control (RB-ANOVA); *n* = 4.

The effect of serially diluted extracts obtained from polymeric nonwovens containing PTX on 4T1 cell growth is shown in Figure 12. These media exhibited highly cytostatic activity against the breast cancer cell line, and the maximal inhibition of cell proliferation occurred at the concentration of 6.25% for both analyzed extracts (higher concentrations of extracts did not cause stronger growth inhibition; therefore, they are not included in the chart). The extract obtained from PDLGA 48:52/PVA + PTX ceased to exhibit cytostatic activity at a concentration of 0.391%. Surprisingly, the nonwoven obtained from PDLGA 48:52 + PTX exhibited greater bioactivity, as significant inhibition of cell proliferation was detected at all tested extract concentrations down to 0.195%.

**Figure 13:**
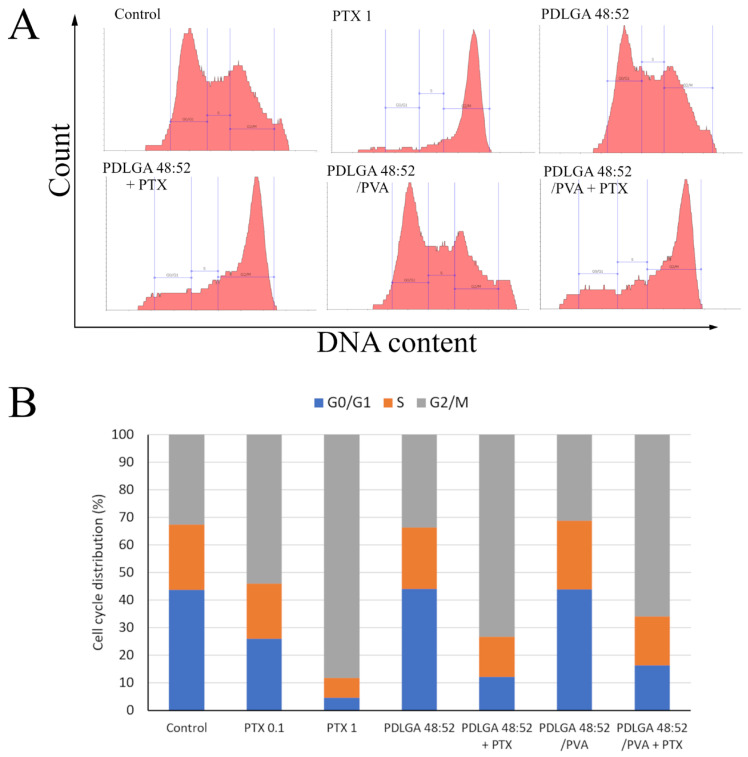
Flow cytometry analysis of the 4T1 cell cycle. Cells were treated with nonwoven extracts at a concentration of 12.5% or with PTX solutions (at concentrations of 0.1 and 1 µg/mL) for 24 h. (**A**) Representative histograms displaying DNA content; (**B**) calculated proportions of cells in the G0/G1, S, and G2/M phases of the cell cycle; *n* = 3.

To determine whether the reduced cell growth rate was caused by disturbances in cell cycle progression, the cell cycle phase profile was determined using flow cytometry and staining with the Vybrant™ DyeCycle™ Green Stain. In the control, DNA content histograms present a typical pattern, with a dominant G0/G1 phase (Figure 13A). Cell cycle profiles in 4T1 cell cultures incubated with extracts from drug-free nonwovens for 24 h are very similar to those in the control. However, cell treatment with free PTX solutions resulted in powerful alterations in cell cycle distribution. As expected, given the mechanism of the cytostatic action of PTX, an accumulation of cells in the G2/M phase occurred. The number of cells characterized by the presence of replicated DNA increased from approximately 32% (in the control) to 54% and 88% in cultures treated with 0.1 µM and 1 µM PTX, respectively. This proves the blockage of mitotic cell division due to the prevention of proper chromosome segregation. The effects of extracts from nonwovens containing PTX were characterized by identical changes in cell cycle distribution to those observed with free PTX as follows: a significant accumulation of cells in the G2/M phase was observed. Our observations strongly suggest that the inhibition of proliferation in 4T1 cell cultures incubated with these extracts was actually due to PTX released from the nanofibers into the medium. Interestingly, the effect of the drug dosage form made from PDLGA 48:52 + PTX on cell accumulation in the G2/M phase was slightly greater than that observed for PDLGA 48:52/PVA + PTX.

### 2.5. Tumor Growth Inhibition

**Figure 14:**
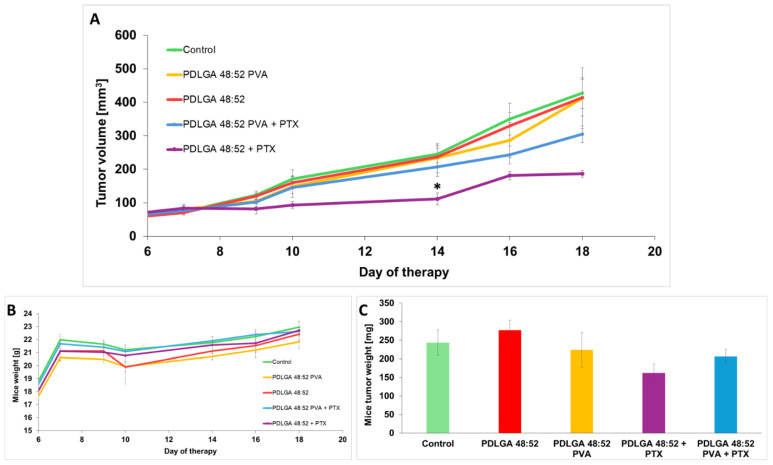
The impact of the electrospun nonwovens carriers on the growth of 4T1 tumors. (**A**) Tumor growth inhibition after the carriers’ implantation directly into the tumors. (**B**) Graph showing the mice’s weight during the experiment. (**C**) Graph showing the tumor weight on the last day of the experiment. The data are shown as the mean ± SEM. Tukey’s multiple comparisons test; * *p* < 0.05.

To evaluate the anticancer efficacy of the tested nonwovens, experiments were performed on the 4T1 mouse breast cancer model. Mice with established breast cancer tumors with a volume of approximately 100 mm^3^ had appropriate nonwovens inserted into the central part of the tumor. The inserts contained drug-free nonwovens (PDLGA 48:52, PDLGA 48:52/PVA) and nonwovens with PTX (PDLGA 48:52 + PTX, PDLGA 48:52/PVA + PTX). In the control group, the tumor was transected as in the other groups. Over the next 11 days, tumor volume was measured for each group. Tumor growth inhibition was observed in the group receiving PDLGA 48:52 + PTX nonwovens. On day 14, tumor volume was 55% smaller than in the control group. In mice implanted with PDLGA 48:52/PVA + PTX nonwovens, tumor volume was 15% smaller than in control tumors. Tumor volume was comparably larger in all control groups (Figure 14A). Meanwhile, no reduction in mouse weight was observed after the administration of nonwovens in any of the tested groups (Figure 14B). The weight of the tumors after their excision on day 18 was the lowest in the group that received PDLGA 48:52 nonwovens with PTX (PDLGA 48:52 + PTX) (Figure 14C). The tumors experience a weight reduction of 34% compared to the control tumors. The other tumors were of similar weight. Furthermore, tumors receiving PDLGA 48:52/PVA + PTX were consistently smaller. The tumors weighed 15% less than the control tumors. However, the differences were not statistically significant.

### 2.6. Morphological Changes and Tumor Cell Proliferation and Apoptosis in 4T1 Tumors

**Figure 15:**
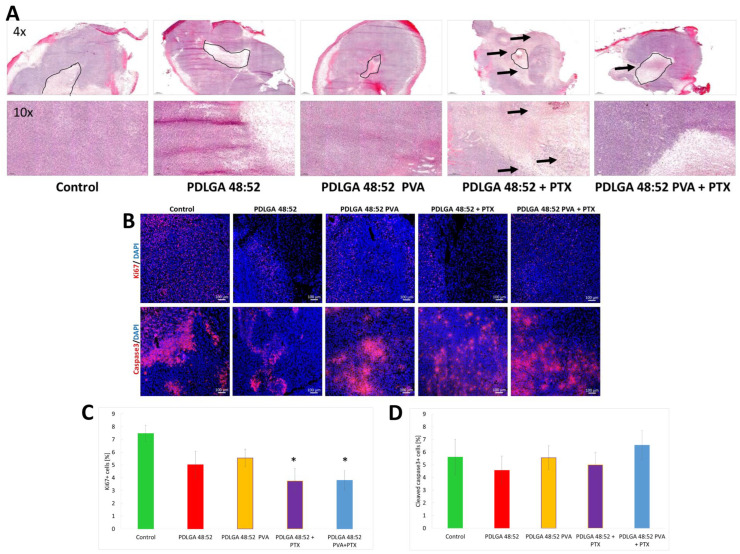
The impact of the electrospun nonwoven carriers on the morphological changes, tumor cell proliferation, and apoptosis in 4T1 tumors. (**A**) Whole slide images (upper panel; 4× magnification) of representative hematoxylin and eosin staining and photographs of tumors at higher magnification (lower panel; 10× magnification) are shown. The location of the implants is marked with a black border in the top panel. The arrows in both panels indicate the location of immune cell infiltration. (**B**) Representative images of Ki67 and cleaved caspase 3 staining. (**C**) Tumor cell proliferation after nonwoven carrier therapy. Images were taken using a confocal microscope (20× magnification). The data represent the percentage of the positively stained area. (**D**) Tumor cell apoptosis after nonwoven carrier therapy. Images were taken using a confocal microscope (20× magnification). The data represent the percentage of the positively stained area. Results are shown as the mean ± SEM. Kruskal–Wallis multiple comparisons; * *p* < 0.05.

Tissues were collected on the 18th day and sections were prepared, in which the tumor structure was stained using H + E staining. The sections showed increased areas of cell death and immune cell infiltration in the region where PDLGA 48:52 + PTX nonwovens were introduced (Figure 15A). In groups of mice in which control nonwovens (PDLGA 48:52 and PDLGA 48:52/PVA) were introduced into the tumor, and in control tumors, no increase in cell death or infiltration of immune system cells within the implant were observed. Nevertheless, in the group of mice that received PDLGA 48:52/PVA + PTX nonwovens, an increase in cell mortality in the vicinity of the implant and the infiltration of immune system cells were observed. However, this effect was significantly smaller than in the group of mice that received PDLGA 48:52 + PTX nonwovens (Figure 15A). Tumor tissue destruction and immune cell infiltration were significantly further away from the implant in the group of mice that received PDLGA 48:52 + PTX implants. In the group of mice that received PDLGA 48:52/PVA + PTX implants into the tumor, this effect was observed in close proximity to the implant. The number of proliferating cells was determined in the obtained tumor preparations by Ki67 staining. The results indicate reduced proliferation in groups that received nonwovens with PTX, both the PDLGA 48:52 and PDLGA 48:52/PVA (Figure 15B,C). In both cases, a 50% decrease in the number of proliferating cells was observed compared to the control group. No statistically significant difference was observed between the groups that received empty nonwovens and the control group. As PTX causes caspase-dependent apoptosis, the presence of cleaved caspase 3 was determined in the specimens. However, the data do not indicate an increase in caspase 3 cleavage in the tested tumor sections (Figure 15B,D).

## 3. Discussion

PTX is a chemotherapeutic agent that belongs to the taxane family, approved to treat various kinds of cancers [32]. Although PTX has been used for two decades, either as a single drug or in combination with other chemotherapeutics, it may cause serious adverse drug effects [32,33]. Therefore, PTX-loaded biodegradable polymeric implants may be an alternative solution and could provide local delivery of the active agent to malignant cells in a continuous, sustained, and predictable manner, avoiding toxic chemotherapy’s adverse effects [32]. Additionally, the postsurgical local insertion of a biodegradable implant device loaded with an anticancer drug can prevent the further spread of cancer cells [34].

Electrospun membranes loaded with anticancer drugs have been broadly studied, and many of them present promising therapeutic effects on cancer cell inhibition, tumor size reduction, and the life extension of tumor-bearing animals [18,35].

However, their drug release profiles are difficult to predict, since degradation patterns may vary for crystalline polymers. Therefore, there is a need to improve and optimize the release process and achieve better therapeutic outcomes [35]. The aim of our study was to analyze the possibility to tailor the release of PDLGA ES nonwovens through the choice of polymer, its comonomer molar ratio, and molar mass. PDLGA is a widely explored US FDA-approved biocompatible polymer that combines the advantages of both PLA and PGA [36]. The main advantage of the aliphatic polyesters is their biocompatibility and hydrolytic degradation to the products that are naturally present in the human body. The degradation rate is easily tailorable (e.g., by molar mass, composition, and copolymer microstructure). These features may be adjusted at the synthesis level by the choice of the appropriate initiator for the copolymerization reaction, the time and temperature of the polymerization, and the molar ratio of the comonomers.

In our study three kinds of polymers were compared with a lactidyl-to-glycolidyl comonomer ratio of 86:14, 70:30, and 48:52. Also, nonwoven obtained from a blend of PDLGA 48:52 and PVA was used to analyze the effect of the addition of the hydrophilic polymer on degradation and, thus, drug release rate. The PTX-loaded nonwovens were successfully obtained by the electrospinning method. Electrospun membranes could be fabricated using various combinations of parameters (method of solution preparation, fabrication techniques, drug types/loading methods, etc.), which may affect the drug release rate and profile [35]. Also, the solubility and compatibility of the drugs with the drug/polymer/solvent system is an important factor that must be considered in the electrospinning process [37]. In fact, the parameters for developing various nonwovens from PDLGA have been adjusted and optimized individually for each kind of polymer (Table 6). The study revealed that all kinds of polymers were suitable for processing by the electrospinning method and formed nonwovens with a smooth surface of fibers without pores (Figure 3). Also, the PTX was successfully incorporated into the nonwovens, as visualized in the NMR spectra (Figure 2).

The nonwovens were subjected to in vitro analysis of degradation and PTX release. Generally, device behavior in terms of both degradation and release characteristics is the result of the synergic effect of several phenomena [38]. The degradation of PLGA occurs via the hydrolysis of ester bonds to products that are naturally present in the human body (e.g., lactic acid and glycolic acid) [19]. Initially, water permeates the amorphous regions of the polymer matrix, breaking the ester bonds. In turn, the molar mass of the polymers is reduced, which raises their hydrophilicity and speeds up the breakdown of the polymers into water-soluble fragments. Finally, these fragments are hydrolyzed into lactic acid and glycolic acid, which are then broken down by normal metabolic pathways into energy, CO_2_, and water [39,40,41,42]. The analyzed ES nonwovens exhibited a gradual decrease in weight (Figure 4) and molar mass (Figure 5) and changes in comonomer unit ratio (Table 3) and the T_g_ (Table 4). It can be observed that the changes in the polymers during degradation strongly correlated with drug release. It should be also underlined the all the physicochemical changes in the polymers proceeded gradually, which facilitated regular drug release (Figure 10B). The composition of PDLGA affects the rate of degradation and, therefore, the kinetics of drug release. Generally, the degradation of poly(D,L-lactide-co-glycolide) accelerates with the increase in glycolidyl unit content [43,44,45,46]. In fact, the degradation of the nonwoven depended on its polymeric composition and molar mass. According to our expectations, the PDLGA 86:14 + PTX demonstrated the highest content of lactidyl units, and its molar mass presented the highest stability during incubation. However, the slow degradation process resulted in very limited PTX release (Figure 10). It has been determined that the release of PTX from PDLGA 86:14 + PTX was controlled mainly by the polymer (anomalous transport) (Table 5). The polymer degradation rate increased with the increase in glycolidyl unit content in the copolymer and was the fastest for PDLGA 48:52/PVA + PTX. The acceleration of the degradation also increased the rate of the drug release. In the case of the other three types of nonwovens, Fickian diffusion became the dominant mechanism of drug release with the increase in glycolidyl unit content in the copolymer (Table 5). The highest influence of Fickian diffusion was observed for PDLGA 86:14/PVA + PTX. There have been numerous attempts to understand the mechanism of drug release through nanofibers, and it has been concluded that it is the result of various factors (the physicochemical properties of the drug, the composition and structure of the polymeric matrix, the release conditions, and the possible interactions among all these factors) [47]. It has been determined that the key mechanisms involved in the drug release from polymeric nanofibers involve drug diffusion, polymer matrix swelling, and material degradation [47]. Another important factor that must be considered, especially in the case of poorly water-soluble drugs, is the release environment, e.g., the selection of the release medium and volume that provide the sink conditions. Initially, we conducted the release study in PBS and maintained sink conditions by regularly exchanging the release medium. However, PTX release was very limited in the first phase (first days or even weeks) (Figure 10A). Therefore, the study was repeated in PBS with Tween 80 (0.5%), which is a commonly used surfactant for the release of poorly water-soluble drugs [48,49]. The change in release medium resulted in the acceleration of the release rate and made the release profiles more regular (Figure 10B) compared to the PBS. This may be indicative that PBS with Tween 80 as a surfactant provides better sink conditions for PTX release.

The most commonly produced and commercially available copolymers have a lactide/glycolide ratio of 85:15, 75:25, and 50:50. Therefore, changes other than the comonomers’ unit ratio must be applied to accelerate the degradation of PLGA 48:52. We hypothesized that blending this copolymer with hydrophilic polymer, e.g., PVA, may be a solution. Although the degradations of PDLGA 48:52 + PTX and PDGLA 48:52/PVA + PTX were similar, the PTX release from PDLGA 48:52/PVA + PTX was significantly faster (Figure 10). This may be caused by the increase in the hydrophilicity of the nonwoven obtained by blending the PDLGA with PVA that was confirmed by contact angle measurement (Table 1). This effect probably facilitated enhanced drug diffusion. It is known that polymer hydrophilicity/hydrophobicity determines the water uptake and the rate of hydrolysis. Hydrophilic and amorphous materials can retain a greater amount of water and are thus subjected to a faster degradation [38]. It has been observed that the contact angle of PDLGA 48:52/PVA + PTX is lower compared to that of PDLGA 48:52 + PTX (Table 1), and PVA remains in the nonwoven even after 28 days of degradation, which was visualized in GPC chromatograms (Figure 6). It has been already reported that the incorporation of PEG may enhance the release kinetics of PTX from PLA fibers [23]. However, the effect of blending PDLGA with PVA to increase the release rate of PTX has not been studied so far. Our study clearly demonstrates that the developed blend may efficiently support an increase in drug release. Importantly, the release process from PDLGA 48:52/PVA + PTX is not rapid but extended over 28 days.

The in vitro experiments were conducted to analyze the cytotoxic effect of PTX released from PDLGA 48:52 + PTX and PDLGA 48:52/PVA + PTX nonwovens against the 4T1 murine mammary carcinoma cell line (Figure 12 and Figure 13). In this experiment, complete cell culture medium, a fluid with a highly complex composition, was used as the extraction fluid. It contained 10% FBS, a reagent rich in albumin proteins with a high affinity for PTX. Therefore, it can be hypothesized that, under such conditions, the release of PTX from the nonwovens could have been significantly more efficient than in the case of PBS. It can be assumed that such extraction conditions reflect relatively well the situation in the body. Although the extract obtained from both materials exhibited cytostatic activity, the nonwoven obtained from PDLGA 48:52 + PTX showed greater bioactivity because significant cell proliferation inhibition was detected at all tested extract concentrations at lower concentrations (0.195%) compared to the extract obtained from PDLGA 48:52/PVA + PTX (0.781%) (Figure 12). The same phenomena were observed in flow cytometry analysis, which showed an accumulation of cells incubated with the extracts obtained from both kinds of nonwovens in the G2/M phase, but the effect was slightly greater for PDLGA 48:52 + PTX (Figure 13). This is a result of lower dose of PTX in PDLGA 48:52/PVA + PTX nonwoven, caused by its thinner structure and lower weight compared to PDLGA 48:52. Although the drug dose in 1 mg of nonwoven was similar for both kinds of nonwovens (Table 1), the lower weight of 5 mm discs used in the study resulted in almost half the PTX dose in PDLGA 48:52/PVA + PTX (257.4 µg vs. 404.1 µg).

The data obtained by the in vivo study in the 4T1 mammary carcinoma model indicated tumor growth inhibition only in mice that received PDLGA 48:52 + PTX nonwovens. However, in mice that received PDLGA 48:52 PVA + PTX implants, tumor growth was not as strongly inhibited. This effect was probably also caused by the lower PTX dose in 3 mm discs (78.3 µg vs. 134.3 µg in PDLGA 48:52 + PTX and PDLGA 48:52/PVA + PTX, respectively). Immunohistochemical staining showed increased tumor cell death in tumors in which PDLGA 48:52 + PTX discs were introduced. In addition, the increased infiltration of immune system cells was observed in the areas surrounding the implant. Further studies showed a reduction in the number of proliferating cells in tumors where PTX implants were placed, both PDLGA 48:52 + PTX and PDLGA 48:52 PVA + PTX. However, although PTX induces the apoptosis of cancer cells associated with caspase 3 activation, no increase in its amount was observed in the tumors.

In vivo studies confirm the effectiveness of the constructed nonwovens, especially PDLGA 48:52 + PTX. These results are consistent with other published data on PTX releasing ES nonwovens designed for local therapy. The efficacy of PTX delivered in ES nanofibers obtained from poly(D,L-lactide-co-glycolide) (PDLGA) 50:50 against triple-negative breast cancer progression was confirmed in a mouse model [15]. However, it seems that for fast-growing tumors the construction of nonwovens from which the drug is released more quickly should inhibit tumor growth even more effectively. In our opinion, the use of our nonwovens in people whose tumors grow significantly slower should be effective in combating cancer. It is also important to remember that one of the advantages of engineered nonwovens is that their size can be adjusted to fit the tumor, which should personalize their use for effective cancer treatment.

## 4. Materials and Methods

### 4.1. Chemicals

Monomers: D,L-lactide and glycolide were purchased from HUIZHOU Foryou Medical Devices Co. Ltd. (Huizhou, China). Paclitaxel (PTX) was obtained from MedChemExpress EU (Sollentuna, Sweden). Hexafluoroisopropanol (HFIP) was obtained from Fluorochem Ltd. (Hardfield, UK). Chloroform was purchased from Avantor Performance Materials Poland S.A. (Gliwice, Poland). Methanol was obtained from Glob Center (Piastów, Poland). Fetal bovine serum (FBS), HEPES (4-(2-hydroxyethyl)-1-piperazineethanesulfonic acid), and Vybrant™ DyeCycle™ Green Stain were purchased from Thermo Fisher Scientific (Waltham, MA, USA). Poly(vinyl alcohol) (PVA, average M_w_ 31,000–50,000, 87–89% hydrolyzed) and other reagents not listed above were purchased from Merck Life Science/Sigma-Aldrich (Poznan, Poland).

### 4.2. Synthesis of Polymers

Three different compositions of poly(D,L-lactide-co-glycolide) (PDLGA) were synthesized with the following ratios of lactidyl to glycolidyl units: 86:14, 70:30, and 48:52. The PDLGA was synthesized in bulk by ring-opening polymerization (ROP) of D,L-lactide and glycolide using zirconium (IV) acetylacetonate as a non-toxic initiator. The process involved melting the monomers at 160 °C, followed by heating at 130 °C for 72 h. The obtained materials were purified by dissolution in chloroform and precipitation in cold methanol. The purified polymers were dried in a vacuum at room temperature and then under decreased pressure (80 mbar) to achieve a constant weight.

### 4.3. Preparation of ES Nonwovens

Four types of PTX-loaded nonwovens were produced by the electrospinning method as follows: composed of PDLGA 86:14, PDLGA 70:30, PDLGA 48:52, or a mixture of PDLGA 48:52 with PVA. PDLGA nonwoven was made from a solution of a copolymer in a mixture of solvents (15% *w*/*w* for solution of PDLGA 86:14 and PDLGA 70:30, 20% *w*/*w* for solution of PDLGA 48:52). The solvent mixture was composed of chloroform and HFIP in a volume ratio of 4:1. The PDLGA/PVA nonwoven was obtained by using only HFIP as a solvent (16% *w*/*w*) with a PDLGA and PVA mass ratio of 9:1. In the first step, PVA was dissolved in HFIP at 48 °C and stirred on a magnetic stirrer at 350 rpm. PDLGA was added after complete dissolution of PVA and the PDLGA/PVA mixture was left on a magnetic stirrer at 350 rpm overnight.

The nonwovens were obtained with a 5% (*w*/*w*) concentration of PTX using a TL-Pro-BM electrospinning unit (Tong Li Tech, Shenzen, China). The device was equipped with two high-voltage power supplies. A positive electrical potential was applied to the spinneret while a negative potential was applied to the fiber collector in the form of a steel arbor with a 20 mm diameter. Solutions of the copolymer were administered to the spinning nozzle through a PTFE capillary, using a PHD Ultra 4400 syringe pump (Harvard Apparatus, Holliston, MA, USA). The distance between the nozzle and the collector was 25 cm and the G20 nozzle size was used. The 20 mm collector rotated at 500 rpm. The detailed description of parameters used in the production of nonwovens is presented in Table 6. The production was conducted in a clean room ISO 7 (ISO 14644-1) standard. To provide complete solvent evaporation, the nonwovens were air-dried for 3 days at 25 °C and dried for 21 days at reduced pressure (105 mbar) (Memmert VO 400; Schwabach, Germany).

**Table 6:** The parameters used in the formation of various types of PDLGA nonwovens with PTX by the electrospinning process.

Parameters of the Electrospinning Process	Type of Nonwovens			
	PDLGA 86:14 + PTX	PDLGA 70:30 + PTX	PDLGA 48:52 + PTX	PDLGA 48:52/PVA + PTX
Voltage + [kV]	13.5	12.5	10.7	8.5
Voltage − [kV]	8.0	10.0	9.4	6.6
Volume of dispensed solution [mL]	11.0	11.5	11.0	16.0
Solution dispensing speed [mL/h]	1.5	1.5	1.5	2.0
Nonwoven width [cm]	16.0	16.0	15.5	22.0
Temperature [°C] [chamber/ambient]	20.9/20.8	20.0/20.2	20.5/20.3	21.4/20.4
Humidity [%] [chamber/ambient]	34/41	35/40	41/35	41/46

### 4.4. Characteristics of the Polymer and Nonwovens

The polymer was analyzed after synthesis, processing, and degradation by means of gel permeation chromatography (GPC), nuclear magnetic resonance spectroscopy (NMR), and differential scanning calorimetry (DSC).

Proton nuclear magnetic resonance (^1^H NMR) spectroscopy (Avance II Ultrashield Plus 600 MHz, Bruker, Billerica, MA, USA) was used to analyze the comonomer unit’s ratio. The ^1^H NMR spectra were obtained at 22 °C with 64 scans, 1 s acquisition time and 11 µs pulse. DMSO-d6 was used as a deuterated solvent. Chemical shifts (d) were given in ppm using tetramethyl silane as an internal reference.

The number-average molar mass (M_n_) and weight-average molar mass (M_w_) were determined using GPC (Spectra Physics SP 8800 chromatograph, Milpitas, CA, USA).

A DSC (Q2000, TA Instruments, New Castle, DE, USA) was used to determine the thermal properties of the material. The instrument was calibrated using high-purity indium. The samples were heated from 0 to 220 °C under a nitrogen atmosphere (flow rate: 50 mL/min) at a heating rate of 20 °C/min. The copolymer’s melting temperature (T_m_) was determined from the first heating run as the peak maximum of the melting endotherm. The glass transition temperature (T_g_) was measured as the midpoint of the change in heat capacity of the amorphous sample after it was quenched from melting by liquid nitrogen.

Scanning electron microscopy (SEM, Quanta 250 FEG, FEI Company, Hillsboro, OR, USA) was used to analyze the morphology of the nonwoven. The analysis was conducted under low vacuum conditions (80 Pa) and with an acceleration voltage of 5 kV from the secondary electron collected by a Large Field Detector. For each sample, images were taken at a magnification of 500 to 5000 times. The average diameter of the fibers in the samples was evaluated by using ImageJ software based on the measurement of 100 fibers. The average diameter of the fibers in the samples was evaluated by using ImageJ software based on the measurement of 100 fibers.

### 4.5. In Vitro Degradation Study

For the analysis of the in vitro degradation, the nonwovens were cut into discs with a diameter of 10 mm and incubated in 5 mL of PBS (pH 7.4) at 37 °C under constant agitation at 350 rpm. At predetermined intervals (0, 7, 14, 28, 56, 84, and 168 days), the samples were collected for further analysis. The buffer was replaced regularly to prevent the accumulation of degradation products.

The degradation rate was analyzed based on the percentage of weight loss (*W_L_*%), which was calculated according to the following equation: (*W_L_*%) = [(*W*_0_ − *W_dry_*)/*W*_0_] × 100, where *W*_0_ is the initial weight of the sample and *W_dry_* is the residual weight of the materials dried under vacuum until a constant weight is achieved. The SEM, GPC, NMR, and DSC analyses were conducted to evaluate changes in the morphology, molar mass, comonomer composition, and thermal properties of the copolymers.

### 4.6. In Vitro Drug Release Study

The release of PTX from discs with a diameter of 10 mm was evaluated under in vitro conditions at 37 °C and pH 7.4 for 24 weeks in PBS and for 4 weeks in PBS with Tween 80 (0.5%; *v*/*v*) [48]. At predetermined intervals (0, 1, 7, 14, 28, 56, 84, and 168 days), the buffer was replaced to maintain the sink conditions. At the same time, the selected samples (discs) were collected in order to evaluate the remaining drug. The amount of PTX remaining in the disc was determined by dissolving the disc in acetonitrile [50]. The samples were centrifuged at 10,000 rpm for 5 min, and the supernatants were filtered through syringe filters of 0.45 µm and evaluated by means of high-performance liquid chromatography (HPLC) (VWR Hitachi/LaChromElite^®,^ Gdansk, Poland) equipped with a LiChrospher^®^ RP-18 column (250 mm × 4 mm, 5 μm) and LiChrospher^®^ RP-18 guard column (4 mm × 4 mm, 5 μm) (VWR, Gdansk, Poland). The mobile phase consisted of acetonitrile and water (60:40, *v*/*v*) delivered at a flow rate of 1 mL/min. PTX was detected at a wavelength of 227 nm.

The kinetics of the drug release process were analyzed by fitting the release profiles to time-dependent equations using DDsolver, an Excel add-in program [51]. The following three mathematical models were applied to determine the PTX release rates from the nonwovens: the Higuchi, Korsmeyer–Peppas, and Peppas–Sahlin models. The Higuchi model is described by the following equation: $M_{t}=K_{H}\cdot t^{1/2}$, where $M_{t\;}$ is the cumulative amount of drug released at time t, $K_{H}\;$ is the Higuchi dissolution constant, and kis the rate constant. The Korsmeyer–Peppas model is expressed as follows: $M_{t}/M_{\infty }=K_{kp}\cdot t^{n}$, where $M_{t}/M_{\infty }$ is the fraction of drug released at time t, $M_{t}$ is the cumulative amount of drug released in time t, $M_{\infty }$ is the total amount of drug released at equilibrium (or after infinite time), $K_{kp}$ is the Korsmeyer–Peppas release rate constant, and n is the diffusional exponent (or release exponent) [30,52]. The Peppas–Sahlin model is represented by the following equation: $F=k_{1}t^{m}+k_{2}t^{2m}$, where $k_{1}\;$ and $k_{2}\;$ are rate constants related to Fickian and non-Fickian (anomalous) transport kinetics, respectively; m  is the diffusional exponent dependent on the device geometry; and F represents the cumulative fraction of drug released [31]. The optimal fitting model was determined based on the coefficient of determination ($R^{2}$) value.

### 4.7. In Vitro Cytotoxicity Study

The 4T1 murine mammary carcinoma cell line (ATCC^®^ CRL-2539) used in this research was purchased from the American Type Culture Collection (ATCC, Manassas, VA, USA). The cells were cultured in RPMI-1640 medium supplemented with 10% fetal bovine serum, 20 mM HEPES, 100 U/mL penicillin, and 100 μg/mL streptomycin. They were maintained at 37 °C in a humidified atmosphere containing 5% CO_2_, supplied by the MCO-19AICUV-PE CO_2_ incubator (Panasonic Healthcare, Osaka, Japan). They were passaged with 0.25% Trypsin-0.5 mM EDTA.

To assess the cytotoxic activity of ES nonwovens with PTX, extracts from the nonwovens were prepared. Simultaneously, the cytotoxicity of the extracts obtained from drug-free ES nonwovens, as well as solutions of free PTX in culture medium (acting as a positive control), was studied. To prepare the extracts, the specimens with 5 mm diameter were cut out from the ES nonwovens and placed in 5 mL of the complete culture medium. The drug dose in 5 mm discs obtained from PDLGA 48:52 + PTX and PDLGA 48:52/PVA + PTX was 404.1 µg and 257.4 µg, respectively. Disks were incubated in medium for 24 h at 37 °C with constant agitation (240 rpm). Extracts of the PTX-loaded nonwovens were serially diluted (1:1, *v*/*v*) with fresh culture medium. The cytotoxic activity of the extracts was evaluated by determining the cell growth inhibition using the In Vitro Toxicology Assay Kit, sulforhodamine B (SRB)-based, according to the manufacturer’s instruction. To exclude any possible negative impact of 24 h incubation at 37 °C on the properties of the culture medium, an additional control group was established in which the medium was incubated without polymer samples.

The 4T1 cells were seeded in 96-well plates (Greiner Bio-One, Kremsmünster, Austria) at an initial density of 2 × 10^3^ cells per well in 100 µL of culture medium. Before exposure to the test agents, cells were cultured for 24 h to enable attachment and initial growth. Then, the culture medium was removed and replaced with the extracts or PTX solutions, followed by a 72 h incubation. At the end of the incubation period, the media were removed from the wells, and the cells were fixed at 4 °C with a 10% trichloroacetic acid solution. Cell growth was then assessed using the SRB assay. The SRB absorbance, solubilized in 200 µL of 10 mM tris(hydroxymethyl)aminomethane solution, was read at 490 nm and 690 nm (reference wavelength) using the MRX Revelation plate reader (Dynex Technologies, Chantilly, VA, USA). Data are presented as the mean ± SD of results from four independent experiments. They were statistically analyzed using randomized block ANOVA (RB-ANOVA), followed by Tukey’s post hoc test. Analysis was performed using Statistica 13.1 software (StatSoft, Tulsa, OK, USA).

To study the cell cycle distribution, 4T1 cells were plated into tissue culture dishes (21.5 cm^2^, Corning, Corning, NY, USA) at an initial density of 2 × 10^5^ cells per dish in 5 mL of culture medium and allowed to attach and grow for 24 h. The cells were treated with extracts and PTX solutions for the next 24 h. Extracts from PTX containing ES nonwovens were diluted at a 1:8 ratio. The cell cycle distribution was studied using Vybrant™ DyeCycle™ Green Stain (Ex/Em = 488/520 nm) (ThermoFisher Scientific, Warsaw, Poland), a DNA intercalating agent that exhibits strong fluorescence enhancement upon binding to DNA. This fluorochrome is intended for the vital staining of cells. Living cells were suspended in medium containing the stain (final concentration of 5 µM) at a density of 2 × 10^5^ cells/mL and incubated at 37 °C for 30 min, protected from light. Finally, the cells were analyzed using the Guava easyCyte 6-2L flow cytometer (Merck, Darmstadt, Germany), and data were processed with guavaSoft™ 4.5.25 software, as well as with Flowing Software 2.5.1. (Perttu Terho, Turku, Centre for Biotechnology, University of Turku, Finland).

### 4.8. In Vivo Study

All animal experiments were conducted in accordance with the National Institutes of Health Guide for the Care and Use of Laboratory Animals and the 3R principles, with approval from the Local Ethics Commission for Animal Experiments in Katowice (permission 13/2025 of 14 February 2025). The drug dose in 3 mm discs obtained from PDLGA 48:52 + PTX and PDLGA 48:52/PVA + PTX was 134.4 µg and 78.3 µg, respectively.

#### 4.8.1. Mice and Ethics Statement

Experiments on animals were carried out according to the National Institutes of Health recommendations and the 3R rules, with the consent of the Local Ethical Committee for Experiments on Animals at the Medical University of Silesia in Katowice (decisions No. 13/2025). The specific information about the animals’ housing and ethics statement are available in the Supplemental Materials and ARRIVE reporting checklist. The mice were housed in the Maria Skłodowska-Curie National Research Institute of Oncology, Gliwice Branch (Poland), in a pathogen-free facility (SPF standard) in a HEPA-filtered Allentown’s IVC system. The mice received a total pathogen-free standard and complete diet (Altromin International, Lage, Germany), water ad libitum, and were monitored daily. The cages were equipped with enrichment elements in the form of cardboard houses and nesting material. Experiments were conducted on female BALB/c mice (8 to 10 weeks old), obtained from Charles River Breeding Laboratories (Wilmington, MA, USA). The inclusion criterion was a developed tumor. The number of animals in each group was kept to a minimum while maintaining the statistical significance of the obtained results. The sample size was estimated for analysis using a one-way ANOVA test with the following assumptions: a statistical significance level of α = 0.05; a statistical power of 1β = 0.8; expected effect size = 0.6; at least 2 independent experiments were performed; *n* = 5 mice per group, per experiment.

#### 4.8.2. Therapy of Mice with the Use of Electrospun Nonwovens

Murine 4T1 cells were injected subcutaneously (2 × 10^5^ cells, lower flank injection). Mice were randomly divided into experimental groups. The following groups were created: (1) control—in which mice did not receive any carriers but the tumors were intersected as in tumors that received nonwovens; (2) PDLGA 48:52—in which mice received a drug-free PDLGA 48:52 carrier; (3) PDLGA 48:52/PVA—in which mice received a drug-free PDLGA 48:52/PVA carrier; (4) PDLGA 48:52 + PTX—in which mice received paclitaxel in a PDLGA 48:52 carrier; and (5) PDLGA 48:52/PVA + PTX—in which mice received paclitaxel in a PDLGA 48:52/PVA carrier. There were 5 mice in each group, and the experiment was repeated twice. Seven days post cancer cell inoculation, when the tumor volume reached about 80–100 mm^3^, the mice were anesthetized with 5% isoflurane (Baxter) and discs with a diameter of 3 mm were inserted directly into the center of the tumors. For this purpose, a 2–3 mm lateral incision was made in the skin, followed by an incision in the tumor and the insertion of the implant into the center of the tumor. The skin of the mice was stitched with surgical sutures. The drug dose in 3 mm discs obtained from PDLGA 48:52 + PTX and PDLGA 48:52/PVA + PTX was 134.4 µg and 78.3 µg, respectively. The tumors were measured with calipers, and tumor volumes were determined using the following formula: volume = width^2^ × length × 0.52. During the experiment, the mice were weighed regularly, and their behavior was observed, which indicated their condition.

#### 4.8.3. Tumor Collection, Immunohistochemistry, and Immunofluorescence Microscope Analysis

On the 11th day after discs were inserted into the tumors, the mice were sacrificed by cervical dislocation. Tumors were collected, embedded in OCT (Leica Biosystems, Wetzlar, Germany), and frozen in liquid nitrogen. Tumors were collected, embedded in OCT (Leica Biosystems, Wetzlar, Germany), and frozen in liquid nitrogen. The frozen tumors were cut into 5 µm sections. The sections were examined histochemically via hematoxylin/eosin staining (Merck, Darmstadt, Germany); the analysis was conducted using the slide scanner (Panoramic 250, 3D Histech, Budapest, Hungary) and processed by SlideViewer software (Version: 2.7). Active caspase 3 was assessed using anti-cleaved caspase 3 antibody (9661, Cell Signaling Technology) and with AlexaFluor594 conjugated secondary antibody (ab150168, Abcam, Waltham, MA, USA). The presence of Ki67 positive cells was assessed using anti-Ki67 antibody (clone: D3B5, 9129, Cell Signaling Technology, Danvers, MA, USA), following AlexaFluor594 conjugated secondary antibody (ab150168, Abcam). Tumor sections were counterstained with DAPI (Merck). Whole tumor sections were scanned (20×). Fluorescence imaging was performed with the confocal microscope LSM710 (Carl Zeiss Microscopy, Jena, Germany) and analyzed with ImageJ 1.48v (NIH, Bethesda, MD, USA).

#### 4.8.4. Statistical Analysis

The results were analyzed using Statistica software version 12 (TIBCO Software Inc., StatSoft Poland, Krakow, Poland). The normality of the distribution was verified with the Shapiro–Wilk test. The homogeneity of variance was checked using the Brown–Forsythe and/or Levene’s tests. Student’s *t*-test or the Mann–Whitney U-test was used to compare two groups of variables. The Kruskal–Wallis test with Dunn’s multiple comparison post hoc test or one-way analysis of variance with appropriate post hoc was used to compare more than two groups of variables. *p*-values < 0.05 were considered statistically significant. The data are shown as the mean ± SEM (Standard Error of Mean).

## 5. Conclusions

The electrospun nonwovens may be used as implantable devices releasing PTX that may enhance therapeutic efficacy by providing local drug release and overcoming the drawbacks of conventional systemic treatment. There have already been some published data demonstrating the great potential and efficacy of ES nonwovens loaded with anticancer drugs. However, there is still a need to develop a method for controlling the drug release rate, which would be helpful in the adjustment of a drug dose for particular therapeutical requirements. Therefore, this study aimed to analyze the possibility to tailor the release process of PTX from poly(D,L-lactide-co-glycolide) (PDLGA) ES nonwoven by selecting polymers with different comonomer molar ratios, molar mass, and hydrophilicity. For this purpose, three kinds of polymers with lactidyl-to-glycolidyl comonomer ratios of 86:14, 70:30, and 48:52 and a blend of PDLGA 48:50 and PVA were compared. It has been observed that the degradations of all kinds of the developed PDLGA nonwovens proceeded in different rates, which also caused variations in the release rates of PTX. The degradation and PTX release rate increase with a decrease in the molar mass and an increase in the glycolidyl unit content in the copolymer. Although PDLGA 48:52 + PTX and PDLGA 48:52/PVA + PTX presented very similar degradation rates, the nonwovens obtained from PDLGA/PVA demonstrated a sufficiently increased drug release rate. Apparently, the increased polymer hydrophilicity improves drug diffusion from the nonwoven. The in vitro and in vivo study conducted in 4T1 mice mammary carcinoma confirmed the biological activity of the released PTX and the efficacy of the developed nonwovens. The results of the study showed the influence of polymer choice in tailoring PTX release from PDLGA ES implants. The findings may be helpful in their translation into the clinic and for better adjustment of the PTX dose for individual treatment.

## Figures and Tables

**Table 1:** Physicochemical properties of nonwovens containing PTX.

Type of Nonwoven	M_n_^1^ [kDa]	M_w_ ^1^ [kDa]	D,L-LA ^2^ [%]	GA ^2^ [%]	T_m_^3^ [°C]	ΔH ^3^ [J/g]	T_g_^3^ [°C]	Contact Angle (θ) [°]	PTX Content [µg/mg]
PDLGA 86:14 + PTX	66.1	139.9	86.0	14.0	83.0	16.1	55.0	-	45.5 ± 1.3
PDLGA 70:30 + PTX	61.0	127.0	70.0	30.0	80.0	28.7	53.0	-	47.9 ± 0.8
PDLGA 48:52 + PTX	29.1	58.1	48.0	52.0	80.0	20.1	49.0	125.7 ^4^ 116.1 ^5^	46.3 ± 1.7
PDLGA 48:52/PVA + PTX	25.0	61.48	48.0	52.0	96.0	13.8	54.0	95.1 ^4^ 84.4 ^5^	45.1 ± 3.0

^1^ M_n_—number-average molar mass, and M_w_—weight-average molar mass, determined by means of gel-permeation chromatography (GPC). ^2^ Molar-% content of lactidyl (D,L-LA) and glycolidyl (GA) units, determined by means of ^1^H NMR spectroscopy. ^3^ Melting temperature (T_m_), the melting endotherm (ΔH), and the glass transition temperature (T_g_), determined by means of differential scanning calorimetry (DSC). ^4^ The inner side (smoother side that was attached to the collector during the production process). ^5^ The outer side.

**Table 2:** Comparison of fiber diameters of produced ES nonwovens (*n* = 100) determined by ImageJ software based on SEM images.

Type of Nonwoven	Mean Diameter [µm]	Min. Value [µm]	Max. Value [µm]
PDLGA 86:14 + PTX	2.90 ± 1.21	1.05	5.46
PDLGA 70:30 + PTX	3.96 ± 2.55	0.33	9.03
PDLGA 48:52 + PTX	3.73 ± 1.45	1.05	6.14
PDLGA 48:52/PVA + PTX	2.74 ± 2.87	0.43	13.96

**Table 3:** Changes in comonomer unit ratio during in vitro degradation of nonwovens determined by means of ^1^H NMR.

Degradation Time [Days]	Ratio of Lactidyl to Glycolidyl Units (D,L-LA:GA)			
	PDLGA 84:16 + PTX	PDLGA 70:30 + PTX	PDLGA 48:52 + PTX	PDLGA 48:52/PVA + PTX
0	84:16	70:30	48:52	48:52
7	84:16	70:30	51:49	49:51
14	84:16	70:30	51:49	50:50
28	84:16	70:30	53:47	51:49
56	84:16	71:29	56:44	54:46
84	85:15	72:28	57:43	60:40
168	85:15	-	-	-

**Table 4:** Changes in the glass transition temperature (T_g_) during the in vitro degradation of nonwovens, determined by means of differential scanning calorimetry (DSC).

Degradation Time [Days]	Glass Transition Temperature (T_g_) [°C]			
	PDLGA 84:16 + PTX	PDLGA 70:30 + PTX	PDLGA 48:52 + PTX	PDLGA 48:52/PVA + PTX
0	55	54	49	55
14	55	53	47	53
28	56	52	49	51
56	54	50	52	50
84	56	52	53	58

**Table 5:** Model parameters of sirolimus release from scaffolds.

Model	Kind of Sample			
	PDLGA 86:14 + PTX	PDLGA 70:30 + PTX	PDLGA 48:52 + PTX	PDLGA 48:52/PVA + PTX
Higuchi				
R^2^	0.8476	0.9416	0.9591	0.9722
Korsmeyer–Peppas				
R^2^	0.9210	0.9541	0.9874	0.9894
*n*	0.909	0.395	0.359	0.383
Peppas–Sahlin				
R^2^	0.9216	0.9575	0.9876	0.9919
k1	−0.171	4.207	10.745	21.355
k2	0.304	1.148	1.195	6.283
m	0.408	0.299	0.306	0.286

## Data Availability

The data presented in this study are available on request from the corresponding author.

## Supplementary Materials

The following supporting information can be downloaded at: https://www.mdpi.com/article/10.3390/ijms262311540/s1.
