# Supplementary material for: Tailoring the Release of Paclitaxel from Electrospun Nonwovens
Source: Int J Mol Sci. 2025 Nov 28;26(23):11540. doi: 10.3390/ijms262311540 (PMC12692280; doi:10.3390/ijms262311540)
Supplement: Supplementary file 1 [file ijms-26-11540-s001.zip › Table S1.pdf]

Table S1. Comparison of fiber diameters of produced ES nonwovens (n=100) determined by ImageJ software based on SEM images.

**Degradation time [day]**	**Mean diameter [μm]**			
	**PDLGA 86:14 + PTX**	**PDLGA 70:30 + PTX**	**PDLGA 48:52 + PTX**	**PDLGA 48:52/PVA + PTX**
**0**	2.900 ± 1.210	3.960 ± 2.550	3.730 ±1.450	2.740 ± 2.870
**1**	2.228 ± 1.078	2.776 ± 1.298	3.411 ± 1.216	5.814 ± 2.420
**7**	2.893 ± 1.227	3.541 ± 1.753	3.111 ± 1.044	5.049 ± 2.783
**14**	2.291 ± 1.774	3.691 ± 1.686	4.272 ± 1.292	ND
**28**	3.124 ± 1.548	3.351 ± 1.746	ND	ND
**56**	3.683 ± 1.775	3.743 ± 1.417	ND	ND
**84**	3.366 ± 1.530	ND	ND	ND
**168**	3.233 ± 1.348	ND	ND	ND

ND – no data - the nonwovens are too degraded to measure the diameter of the fibers.
